# Topics of the Month

**Published:** 1894-12

**Authors:** 


### TOPICS OF THE MONTH.

ONE of the acquisitions which Dr. A. A. Hubbell, of this city, was enabled to make to his ophthalmological library while he was abroad the past Summer, was a "treatise" on diseases of the eye, which is believed to be the first ever published in the English language. It was printed in 1622 and bears the following title:

"A Treatise of One Hundred and Thirteene Diseases of the Eyes and Eye-Liddes. The second time published, with some profitable additions of certaine Principles and experiments, by Richard Banister, Mr. in Chirurgery, Oculist, and Practioner in Physicke.

"God hath created medicines of the earth, and he that is wise, will not contemne them.

"Imprinted at London by Felix Kyngston for Thomas Man, dwelling in Paternoster-row, at the signe of the Talbot. 1622."

This is a duodecimo volume of over 450 pages, and appears to be an effort to counteract, as much as possible, the abuses and charlatanism so prevalent in those days, both by censuring such practices and pointing out the errors of the uneducated and pretentious, and by supplying to the public a work from which might be obtained an intelligent understanding of the anatomy, physiology and diseases of the eye according to the best knowledge of that time.

Banister does not claim that the "treatise" is entirely original with himself, but does not divulge the name of the author whose work he reproduces. In an introductory "to the reader" he expresses his attitude toward the subject and his object in making the publication as follows: "Now to conclude, since the eye is so delicate a member, and of such excellent use, as that without it, we lose most of the best delights and comforts of life; and by it, wee read the Booke of our Redemption, the Roule of times antiquities, the Volumes of the Sages, & the whole Library of the worlds creatures; therefore I could wish, that while it is well, every man were provident enough to perceive it, and when it is ill, cunning enough to cure it. And to shew that I seeke more to bring good to others, than gaine to my selfe; I have here published the labors of a worthy Writer in this kinde, who describeth an hundred and thirteene severall diseases happening to the eyes and eielids, with their particular causes, symptoms, signes, and cures; the worke was dedicated to my former kinsman, and being long since out of print, is not now to be bought for money; and this booke have I sent abroade againe, that those who delight to labor in this art, may runne the readier way to the better successe. I have also added something of mine own, that through my experience they may find at first, what I was learning long."

The book is a very curious and interesting one.

---

THE restriction placed by the state upon the right to practise medicine within its borders is done for the benefit of the people. The sooner this fact is recognized the better it will be for all concerned. The present generation of medical men can derive very little benefit from these wise laws, though it is probable that later generations may do so.

But the public can derive no benefit from the most wholesome laws unless they are properly and adequately enforced. This requires zeal and energy on the part of presecuting officers, and we hope that the young and brilliant district attorney-elect, who has also received the appointment *ad interim* from the governor, will keep a sharp lookout for offenders against our medical practice act.

Physicians, too, have a duty in the premises. They should report all supposed infractions of the law, and thus become a substantial aid to the law officers.

Commenting on this subject, as related to the Maryland practice act, the *Maryland Medical Journal* says:

> Now laws may stand on the statute books and not be enforced, on account of the ignorance on the part of the public and the apathy of the profession. Ignorance of a law is no excuse, but apathy is a greater wrong, and yet many a person sees laws broken and sinned against but is too careless or apathetic to raise objections.
>
> . . . . . . . . . .
>
> It is the duty of every physician in the state to constitute himself a detective and report to the secretary of the State Board of Medical Examiners all persons whom he knows or even suspects to be practising medicine without a right. Physicians have, for a long time, asked for an efficient state medical law, and now one has been passed, it is the duty of respectable physicians to uphold it and do their parts.

---

A BRONZE statue of the late Dr. J. Marion Sims was erected in Bryant park, in the City of New York, on Saturday, October 20, 1894. A description of the statue appeared in the June issue of the JOURNAL, 1894, page 689.

This first tribute in sculpture to a physician in this country had its inspiration in the columns of the New York *Medical Record*. To its editor, Dr. Shrady, is due the securing of a fund sufficient to accomplish the purpose aimed at. At the unveiling ceremonies addresses were made by Drs. George F. Shrady and Paul F. Mundé, after which the concealing veil was drawn by a child, a grandson of Sims, and the statue was formally presented to and accepted by the City of New York.

Dr. Shrady's address was an eloquent tribute to the famous gynecologist whose figure is thus perpetuated in bronze, but whose fame is more enduring than brass or the Egyptian pyramids. Dr. Shrady gave a brief biographical sketch of Sims, and Dr. Mundé told of his struggles in establishing gynecology on a firm foundation and of his organization of the New York Woman's Hospital, and especially how he was selected for the peculiar distinction of having a statue erected to his memory.

The occasion and the participants were all well chosen, and the graceful deed reflects credit on the American profession of medicine.

